# Advances in Miscanthus × Giganteus Planting Techniques May Increase Carbon Uptake in the Establishment Year

**DOI:** 10.1111/gcbb.70012

**Published:** 2024-11-28

**Authors:** Guler Aslan‐Sungur (Rojda), Nic Boersma, Caitlin E. Moore, Emily Heaton, Carl J. Bernacchi, Andy Vanloocke

**Affiliations:** ^1^ Department of Agronomy Iowa State University Ames Iowa USA; ^2^ DOE Center for Advanced Bioenergy and Bioproducts Innovation Iowa State University Ames Iowa USA; ^3^ DOE Center for Advanced Bioenergy and Bioproducts Innovation University of Illinois Urbana Illinois USA; ^4^ School of Agriculture and Environment The University of Western Australia Crawley Western Australia Australia; ^5^ Department of Crop Sciences University of Illinois Urbana Illinois USA

**Keywords:** biomass productivity, carbon uptake, eddy covariance, Miscanthus × giganteus, perennial crops, planting density

## Abstract

Agricultural lands hold significant potential for CO_2_ sequestration, particularly when utilizing biomass crops and agricultural residues. Among these, Miscanthus × giganteus (*mxg*) stands out due to its high productivity and carbon sequestration capabilities. Recognizing the importance of such biomass crops, the Intergovernmental Panel on Climate Change (IPCC) has identified Bioenergy with Carbon Capture and Storage (BECCS) as a crucial strategy for achieving net‐zero CO_2_ emissions by 2050. This study examines the carbon uptake potential of *mxg* during its establishment year at the Sustainable Advanced Bioeconomy Research (SABR) farm in Iowa, USA, where *mxg* was planted at a density exceeding previous studies. Using eddy covariance (EC) measurements, we quantified the net ecosystem carbon exchange (NEE), and derived gross primary productivity (GPP), and ecosystem respiration (*R*_eco_). Our findings reveal that SABR's *mxg* exhibited a significant carbon uptake of −621 g C m^−2^, a threefold increase compared to a similar EC site in the “corn‐belt” (University of Illinois Energy Research Farm; UIEF), which was established with lower planting density and pre‐commercial planting equipment. Favorable growing conditions and advanced planting technologies at SABR likely contributed to this high carbon uptake. Comparisons with other global EC studies indicated a strong correlation between higher planting densities and greater carbon uptake. These results suggest that increasing *mxg* planting density can enhance carbon uptake, but further studies are necessary to evaluate the impacts under varying environmental conditions and management practices. Additionally, economic analyses are essential to determine the viability of higher planting densities. Our study underscores the potential of optimized *mxg* management practices to contribute significantly to CO_2_ uptake and supports the development of BECCS as a viable climate change mitigation strategy.

## Graphical abstract

Miscanthus × giganteus (mxg) shows high potential for carbon sequestration, making it a key biomass crop for Bioenergy with Carbon Capture and Storage (BECCS). This study quantified mxg's carbon uptake during its establishment year at the Sustainable Advanced Bioeconomy Research (SABR) farm in Iowa. Results revealed a carbon uptake of −621 g C m^−2^, three times higher than similar studies, due to increased planting density. Our findings highlight the potential of optimized mxg management to enhance carbon uptake, supporting its role in climate change mitigation.

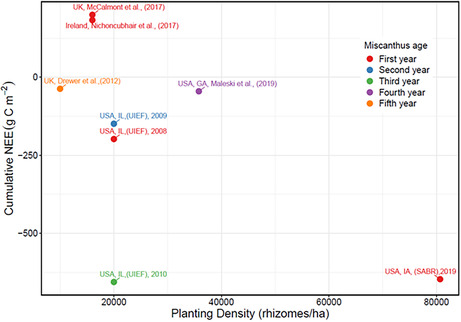

## 1 Introduction

Agricultural lands could be a strong sink for CO_2_, especially when agricultural residues and biomass crops are utilized to offset petroleum use (Burney, Davis, and Lobell 2010; Intergovernmental Panel on Climate Change (IPCC) 2023). Bioenergy with carbon capture and storage (BECCS) plays a pivotal role in the strategies identified to lower global net CO_2_ emissions to net zero by 2050 and limit global warming to 1.5°C (Intergovernmental Panel on Climate Change (IPCC) 2023). Eddy covariance (EC) is a technique that enables us to measure the net ecosystem carbon exchange (NEE) between the ecosystem and the atmosphere, defined as the difference between carbon emission to the atmosphere and uptake by the ecosystem (Baldocchi 2014, 2003). Measurements of carbon uptake using these and other techniques are used to develop and test models that can estimate scenarios at larger scales. With proper land management, models suggest that the cumulative CO_2_ removal (CDR) through BECCS from 2020 to 2100 could be as high as 780 Gt of CO_2_ (Intergovernmental Panel on Climate Change (IPCC) 2023). This equates to an average of approximately 9.75 Gt of CO_2_ annually, representing 25% of estimated fossil emissions for 2023 (Friedlingstein et al. 2023). While promising, management practices that increase CDR in agricultural landscapes through BECCS currently present considerable uncertainty in global carbon budgets (Friedlingstein et al. 2023).

Perennial cropping systems such as Miscanthus × giganteus (Greef et Deu.; hereafter *mxg*) are beneficial in preventing erosion, providing perennial habitat for wildlife, as well and have been shown to sequester more carbon than annual cropping systems (Fraser and Amiro 2013) even during periods with suboptimal growth (Zeri et al. 2011). Furthermore, *mxg* exhibits relatively high yields with very few inputs once established (Lewandowski et al. 2000) which is important to the long‐term carbon balance for this energy crop. Indeed, the carbon balance for this low‐input perennial crop is typically negative leading to increases in soil organic carbon (SOC) suggesting potential for long‐term carbon storage (Brandão, Milà i Canals, and Clift 2011; Jones, Zimmermann, and Clifton‐Brown 2016; Kantola et al. 2022). However, it's important to consider that the carbon dynamics of *mxg* can vary depending on the previous land use. For example, the carbon balance outcomes may differ significantly when *mxg* is planted on land previously used for perennial crops compared to land previously used for annual crops, due to variations in pre‐existing soil carbon stocks and other site‐specific factors. A study by Robertson et al. (2017) found no significant increase in topsoil carbon stocks after 7 years, indicating complexities in soil carbon dynamics. Further, uncertainties surrounding modeled productivity, especially across different growing seasons, suggest additional observational studies are required to confidently estimate *mxg* carbon sequestration potential (Littleton et al. 2023).

The Midwestern United States “corn‐belt” is among the world's most productive agricultural regions (Green et al. 2018; Mueller et al. 2016; Ort and Long 2014). Within the “corn‐belt,” *mxg* is now being grown commercially and has been shown to be a highly productive and profitable perennial biomass crop (Dohleman and Long 2009; Heaton, Dohleman, and Long 2008; Zhang, Sharma, and Khanna 2023). According to the latest information, approximately 20,000 acres of *mxg* are currently being grown across 18 states in the United States (Travis Hedrick, AGgrowtech LLC personal communication). However, among the few studies reporting EC data for *mxg* (Table 1), *mxg* has also been a carbon source during the initial growth phase immediately after planting (i.e., the establishment year). Furthermore, *mxg* is rarely harvested in the first growing season and has been shown to have a highly variable NEE within the first growing season. These challenges impact the early economics and environmental impact of *mxg* production. Changes in management practices that allow for first‐season harvests and increased carbon uptake could improve the early economics and environmental impacts of *mxg* production.

**TABLE 1:** Cumulative NEE with planting density of *mxg* in the literature.

a	Reference	Site	EC tower year	Year/Cumulative NEE (g C m−2)	Planting density (rhizome/ha)
**1**	McCalmont et al. (2017)	Grassland to *M. × giganteus*, Aberystwyth, UK	2012 April	2012/**200**	~16,000/ha
**2**	Ní Choncubhair et al. (2017)	Grassland to *M. × giganteus*, south‐east of IRELAND	2009 April	2009 (April‐Dec)/**183**	16,000/ha
3	Maleski et al. (2019)	Mix Vegetable and row crops to *M. × giganteus*, Georgia, USA	2015 July	2015 (May‐Dec)/**‐45**	35,800/ha
4	Drewer et al. (2011)	Farm (3‐year wheat1‐year oilseed) to *M. × giganteus*, Lincoln, NE ENGLAND	2008 May	2009/‐**37**	10,000/ha
**5**	Zeri et al. (2011)	Arable to *M. × giganteus*, UIEF, IL, USA	2008 July	2008 (Jul‐Dec)/‐**198**	19,000–20,000/ha
**6**	Our study	Arable to *M. × giganteus*, SABR, IA, USA	2019 June	2019 (Jun‐Dec)/**‐621**	80,000/ha

^a^ Dark red numbers refer to the studies reporting first‐year age *mxg* flux data.

*Mxg* is particularly vulnerable to stand failure within the first growing season, and indeed trials reporting *mxg* fields as a net emitter of carbon also exhibited evidence of poor establishment within the first year. For example, McCalmont et al. (2017) reported first‐year peak heights of only 885 mm, and first‐year biomass yields of only 1.4 Mg ha^−1^, which corresponds to approximately 0.63 Mg C ha^−1^ assuming a carbon content of 45%. Additionally, this plot received several herbicide applications, some of which occurred in the third growing season, which is atypical for a well‐established *mxg* stand. McCalmont et al. (2017) also reported yields including weeds, suggesting this stand had significant weed issues, and the *mxg* was likely quite sparse. First‐year NEE at this site was reported as 200 g C m^−2^. Ní Choncubhair et al. (2017) described an even lower first‐year *mxg* stand yield of only 0.10 Mg ha^−1^. This site was shown to be a net source of C with a reported NEE of 183 g C m^−2^. Additionally, this site was not planted until June, significantly reducing the growing season within the first year. Another example of a weakened first‐year stand was reported by Zeri et al. (2011), who described a *mxg* planting event which spanned a time‐period of 2 weeks in June due to adverse weather conditions. The result of this late and prolonged planting was a stand that required additional planting efforts throughout the next two growing seasons to improve stand density (Moore et al. 2021). First‐year, hand‐harvested peak biomass yields averaged only 0.9 Mg ha^−1^ further indicating the effects of adverse conditions at planting. However, despite the challenges of the first growing season, reprocessed data from the *mxg* described by Zeri et al. (2011) showed the most negative NEE (−198 g C m^−2^) for a first‐year stand that we found in the literature. Both McCalmont et al. (2017) and Ní Choncubhair et al. (2017) described grassland conversions to *mxg* while Zeri et al. (2011) reported on a conventional row‐crop transition to *mxg*. All three studies were planted at a rhizome density widely used in both research and commercial applications at the time of establishment ranging from 16,000 (McCalmont et al. 2017; Ní Choncubhair et al. 2017)–20,000 (Zeri et al. 2011) rhizomes ha^−1^ (Table 1).

Historically, *mxg* planting rates have remained steady, between 10,000 and 40,000 rhizomes per hectare, due to the high costs of manual planting (Heaton et al. 2010; Jones 2013; Lewandowski et al. 2000). Although previous research on *mxg* indicates that higher planting density leads to increased productivity (Danatalos, Archontoulis, and Mitsios 2007) and earlier peak yields (Miguez et al. 2008), the yield increases were not thought to offset the cost increases of higher density plantings (Lewandowski et al. 2000). High costs of increased planting density have likely been driven by an historic lack of mechanization of rhizome harvest and planting, and while Miguez et al. (2008) suggest the main advantages to higher planting density will likely occur early in stand development, little is known about the long‐term productivity or economics of increasing planting density.

Furthermore, increased planting density has been shown to significantly impact plant morphology (Zivanovic et al. 2014; Shepherd et al. 2020). However, the effect of planting density may be inconsistent. While Zivanovic et al. (2014) showed a higher number of outgrowths per rhizome in more densely planted *mxg*, Shepherd et al. (2020) reported a lower tiller count in the more densely planted plots. Conversely, Shepherd et al. (2020) showed a positive correlation between planting density and plant height, while Zivanovic et al. (2014) reported shorter plants in more densely planted *mxg* fields. The overall impact of planting density on productivity should be further explored. In recent years, mechanized planting systems have been developed in the United States and the development of fertile, non‐invasive seeded varieties in the UK (Clifton‐Brown et al. 2019; Lewandowski et al. 2016). These advancements have enabled large increases in planting densities, while lowering overall costs per propagule. These technologies have been adopted by commercial operations to ensure early stand success, and while the limited data from establishing *mxg* is unclear whether planting rate strongly affects NEE (Table 1), these recent advancements in technology have allowed for a more than doubling of planting rates (i.e., 80,000 rhizomes/ha).

Crop productivity and carbon fluxes are primarily influenced by environmental factors including light, temperature, precipitation, and soil properties (Baldocchi, Chu, and Reichstein 2018; Wang et al. 2019), as well as management practices (Menefee et al. 2022). Adjustments in crop management, including tillage, fertilization, species selection, and planting density, significantly impact the carbon balance of cropping systems (Davis et al. 2013; Jones, Zimmermann, and Clifton‐Brown 2016; Moore et al. 2020, 2021). Notably, higher planting densities, in some cases, have been linked to increased yields and carbon uptake in various crops (Adams et al. 2015; Licht, Parvej, and Wright 2019), suggesting a potential strategy for enhancing carbon sequestration in croplands. The advancements of the green revolution increased yields of many annual crops through breeding and management, but also increased crop demands for high fertilizer and water (Pingali 2012). As an unimproved sterile perennial crop, not adapted to high inputs, *mxg* will require an alternative approach, particularly optimizing management practices and breeding programs to increase yields (Clifton‐Brown et al. 2019). These breeding efforts, when combined with optimized management, could have the potential to significantly expand *mxg*'s role in carbon sequestration and its contribution to sustainable agriculture.

In this study, we hypothesize that *mxg* planted at higher density, utilizing equipment specifically engineered to harvest and plant rhizomes, would have greater carbon uptake in the first year relative to plots established using legacy equipment and technology such as vegetable transplanters, potato planters, and manually planting. To test this hypothesis, we conducted an EC‐based analysis of the carbon balance of *mxg* grown at the Sustainable Advanced Bioeconomy Research (SABR) farm in Ames, IA, USA that was planted at more than double the planting rate of any other *mxg* EC study (Table 1). Our objectives were to (1) Quantify NEE, GPP, and R_eco_ during the establishment year for *mxg* planted using contemporary commercial planting techniques (SABR), (2) Compare *mxg* establishment year carbon fluxes at SABR to another EC site within the US “corn‐belt” that was established using potato and vegetable transplanting equipment (University of Illinois Energy Research Farm; UIEF), and (3) Contextualize *mxg* NEE at SABR with other EC sites, globally. Our comparison includes data from five sites from four countries with a particular focus on the most proximal site in central Illinois (Moore et al. 2021; Zeri et al. 2011). These objectives help to identify the influence of planting density, a key management practice, on the carbon balance of a clonal perennial crop with implications that include agronomy, life cycle assessment, and policies supporting *mxg* production regionally and globally.

## 2 Methods

To achieve our objectives, we first conducted analysis of data from a newly initiated experiment in central Iowa (Sustainable Advanced Bioeconomy Research; SABR) and then compared those data with historical data from a longstanding experiment in central Illinois (University of Illinois Energy Research Farm; UIEF) and data from previously published studies on *mxg* cultivation in various locations across the USA and the EU (Table 1). We also contextualized the results from the establishment year by including measurements of NEE for maize and *mxg* SABR and UIEF over their respective 3‐year periods which are typically considered the establishment phase for *mxg* (Christian, Riche, and Yates 2008; Shepherd et al. 2020; Tejera et al. 2022). While carbon uptake by *mxg* has been assessed with multiple approaches, for consistency, we limited our analysis to data produced using the eddy covariance (EC) approach.

### 2.1 Site Descriptions

Measurements in *mxg* from the Iowa State University SABR farm (41°59′58″ N, 93°42′15″ W, ~314 m above sea level) began in 2019 and in 2008 at the UIEF (40°3′46″ N, 88°11′46″ W, ~220 m above sea level; Table 2). Both sites have a long history of conventional row‐cropping, predominantly corn‐soy rotations (Bernacchi, Moore, and Pederson 2022; Kantola et al. 2022; Moore et al. 2021). In the immediately preceding growing season, alfalfa was grown at the UIEF site, and soybeans were grown at the SABR farm. While crop type can impact soil respiration which would directly impact NEE, both crops are legumes and have been shown to exhibit similar soil respiration (Adhikari et al. 2023). Both sites comprised fertile agricultural soil that differed in composition. The soil at SABR is primarily Canisteo clay loam (59%), with some Clarion loam (19.3%) and Webster clay loam (18.1%), while at UIEF they are Blackberry silt loam (44.5%), Dana silt loam (41.9%), and Drummer silty clay loam (13.6%) (NRCS 2023; Bohn and Miller, 2024; Tables 3 and 4).

**TABLE 2:** Establishment year management at SABR and UIEF. University of Illinois Energy Farm, Urbana, IL, USA (UIEF). Sustainable Advanced Bioeconomy Research farm, Ames, IA, USA (SABR).

	UIEF			SABR		
Activity	Implement/Product	Rate	Date	Implement/Product	Rate	Date
Tillage	Moldboard plow		Spring 2008	Disc ripper		Spring 2019
Planting	Modified potato planter	~20,000 rhizomes ha^−1^	2‐Jun‐2008 to 16‐Jun‐08	AGgrowTech LLC ACCU DROP Planter	~80,000 rhizomes ha^−1^	3‐May‐19
Herbicide application	Prowl (pendimethalin)	3.5 L ha^−1^	16‐Jun‐08	Harness extra	4.4 L ha^−1^	4‐May‐19
	2,4‐D	1.2 L ha^−1^	16‐Jul‐08	Prowl (pendimethalin)	3.3 L ha^−1^	1‐Jun‐19
				Harness extra	3.3 L ha^−1^	19‐Jun‐19
				Enlist	1.2 L ha^−1^	19‐Jun‐19

**TABLE 3:** Natural resources conservation service (NRCS) web soil survey soil descriptions for SABR and UIEF. University of Illinois Energy Farm, Urbana, IL, USA (UIEF). Sustainable Advanced Bioeconomy Research farm, Ames, IA, USA (SABR).

SABR		UIEF	
Soil description	Plot percentage	Soil description	Plot percentage
Canisteo clay loam, Bemis moraine, 0%–2% slopes	59%	Blackberry silt loam, 2%–5% slopes	44.5%
Clarion loam, Bemis moraine, 2%–6% slopes	19.3%	Dana silt loam, 2%–5% slopes	41.9%
Webster clay loam, Bemis moraine, 0%–2% slopes	18.1%	Drummer silty clay loam, 0%–2% slopes	13.6%
Clarion loam, Bemis moraine, 6%–0% slopes	3.1%		
Nicollet loam, 1%–3% slopes	0.5%		

**TABLE 4:** SABR soil characteristics as measured in Bohn et al., 2024. Sustainable Advanced Bioeconomy Research farm, Ames, IA, USA (SABR).

Soil characteristic	Value (%)	
	SABR	UIEF
Total carbon	1.85	NA
Total organic carbon	1.29	1.71
Organic matter^a^	2.58	3.25^a^
Total nitrogen	0.08	0.15
Clay content	29.8	22
Sand content	36.4	16
Silt	33.3	62

*Note:* NA—Value was unavailable from direct soil sampling at time of establishment.

^a^ Organic matter calculated following Pribyl, 2010.

The climate variables, such as air temperature (*T*_air_ ) and precipitation during the establishment years at both sites, were compared with long‐term data (1991–2020) and reported in Section 3.1. The climate data were retrieved from the Iowa Environmental Mesonet (Iowa State University, 2020). For SABR, we used daily weather data from the Iowa State Agricultural Engineering & Agronomy (AEA) Farm in Boone County, IA, located approximately 10 km from SABR. For UIEF, we used data from Willard Airport in Champaign, IL, located 7.4 km away from UIEF. Long‐term data (1991–2020) were obtained from the National Centers for Environmental Prediction (NCEP: NOAA 2023). The growing degree days (GDD) were calculated using the equation from Tejera and Heaton (2017) with a base temperature of 6°C for *mxg*.

### 2.2 Management

With the exception for planting methodology and rate, management in the establishment year was very similar between SABR and UIEF (Table 2). Briefly, conventional tillage preceded planting, and herbicides were applied to prevent weed growth. Neither site received supplemental nitrogen within the establishment season. *Mxg* was not harvested within the study window during the first growing season. For more detailed information regarding UIEF *mxg* management and establishment, see Kantola et al. (2022); Moore et al. (2021); Smith et al. (2013).

### 2.3 Planting Description

The 3.5 ha plot at SABR was planted on May 3, 2019 using an automated commercial planter (ACCUplanter by AGgrow Tech, High Point, NC, USA in partnership with Spudnik Equipment, Blackfoot, ID). Briefly, the planter created eight parallel furrows spaced 0.61 m apart where at least three rhizomes were dropped in 0.61 m intervals resulting in an upper‐limit planting density of 80,623 rhizomes ha^−1^. SABR rhizomes were harvested sometime between the previous fall and the spring of planting between October 15 and April 15. The 2‐ to 10‐year‐old mother stands were located near Mount Olive, North Carolina, US. Rhizomes were dug, processed, and moved to cold storage within a 3‐day window and were stored for up to 6 months in cool moist conditions which have little impact on emergence (Travis Hedrick, AGgrowtech LLC. personal communication).

Planting at UIEF was accomplished using a modified vegetable planter that achieved an estimated planting density of 20,000 rhizomes ha^−1^ in a 4 ha plot (Smith et al. 2013; Zeri et al. 2011). Above‐average rainfall events throughout the spring and during planting led to a delayed and extended planting window which occurred between June 2, 2008 and June 16, 2008.

### 2.4 Eddy Covariance Instrumentation and Data Analysis

The EC was used to measure ecosystem‐scale carbon fluxes for *mxg* at both the SABR and UIEF sites. The flux towers were situated in the center of the fields as wind conditions were largely omnidirectional at SABR and UIEF (Figure S1). At SABR, the EC tower measurements started on June 01, 2019. The EC system was comprised of an open path infrared gas analyzer (LI‐7500DS, LICOR Biosciences, Lincoln, NE, USA) to determine atmospheric CO_2_ and H_2_O concentrations and a 3D sonic anemometer (GILL WindMaster, Hampshire, UK) to determine sonic temperature, wind speed, and direction. All measurements were recorded at 20 Hz. Supporting meteorological measurements included air temperature (*T*_a_) (HMP‐155 Campbell Scientific, Logan, UT, USA); soil temperature (*T*_soil_) and moisture at 10 cm (Hydra Probe II, Stevens Water Monitoring Systems, Portland, OR, USA). All above ground instruments were initially installed at a height of 2.5 m above the land surface and raised as the crop canopy grew to ensure the instruments were a minimum of 1 m above the canopy at all times. Meteorological measurements were recorded at 1 Hz and averaged at 30‐min intervals. The 20 Hz flux data were processed to 30 min averages using EddyPro software (EddyProv7.00 (SABR), LICOR Biosciences, Lincoln, NE, USA). EddyPro (v7.0.9; LICOR Biosciences) configurations included flux detrending via block averaging, instrument tilt correction with double rotation, and time lag correction through covariance maximization. Flux density correction followed Webb‐Pearman‐Leuning's approach (Webb, Pearman, and Leuning 1980), with spike identification and removal as per Vickers and Mahrt (1997) and footprint calculations from Kljun et al. (2004) for SABR. The data then passed through quality assurance/quality control QA/QC to eliminate spikes. A friction velocity (u*) threshold was established for the site using the moving point test (Papale et al. 2006), which identifies and removes periods of low turbulence that can lead to unreliable flux measurements. Meteorological data for SABR were gap‐filled with external data from the NOAA ISD at Ames Municipal Airport (the site 7.4 km away from the external station). Following this, the u* filter was applied to remove data under poor turbulent transport conditions. Subsequently, carbon, water, and energy fluxes were gap‐filled using the marginal distribution sampling (MDS) technique (Reichstein et al. 2005). Finally, net ecosystem exchange (NEE) was separated into ecosystem respiration (*R*_eco_) and gross primary productivity (GPP) using the nighttime temperature response function by Lloyd and Taylor (1994) for SABR.

Similar to SABR, the EC technique was used at UIEF in Urbana, Illinois, USA, to measure ecosystem carbon flux. The EC measurements over *mxg* at UIEF, which began on July 8, 2008, employed a comparable suite of instruments (Cheng et al. 2020; Moore et al. 2021; Zeri et al. 2011, 2020), as detailed in Table S1. The data processing is extensively explained by Moore et al. (2020). However, unlike SABR, UIEF utilized the LI‐7500RS model of the infrared gas analyzer, recording at 10 Hz. The EddyPro (v6.2.0; LICOR Biosciences) configuration differed only in the footprint calculation, based on Hsieh, Katul, and Chi (2000). Meteorological data for UIEF were gap‐filled with external data from the University of Illinois Willard Airport and surface climatology from ERA5 (Wu et al. 2022). To ensure consistency in gap‐filling and flux partitioning between SABR and UIEF, we used the same method as applied to the SABR data. Similar to SABR, for UIEF data, the MDS gap‐filling method was used, and flux partitioning into Reco and GPP was accomplished by applying the Lloyd and Taylor (1994) method at UIEF.

The cospectral analysis for both sites showed that the heights of the EC towers were sufficient to capture vertical turbulent transport (Figure S3). Additionally, a footprint analysis was performed for both sites using the TOVI tool (LICOR Biosciences), which confirmed that the fluxes originated from the areas of interest at both sites (Figure S2).

### 2.5 Statistical Analysis

Statistical analyses were performed using R software (R Core Team, 2022, version 4.1.2). The comparison of fluxes (NEE, GPP, and *R*_eco_) between the SABR and UIEF sites was conducted using descriptive statistics due to the absence of spatial replication and temporal difference between the sites. We further executed a Random Forest analysis using the “randomForest” package in R to evaluate the relative importance of carbon flux components (*R*_eco_ and GPP) and key environmental variables (air temperature ‐*T*_air_‐, soil temperature ‐*T*_soil_‐, and soil water content ‐SWC‐) in determining NEE. The Random Forest method, a widely recognized approach in ecological research (Breiman 2001; Moore et al. 2018; Zeng et al. 2020; Zhu et al. 2022; Hutley et al. 2022), involves creating an ensemble of decision trees to show the impact of any variables on the measured data of interest. The Random Forest analysis was conducted independently for SABR and UIEF sites to identify the factors with the most substantial impact on carbon uptake. For each site, the effectiveness of individual environmental variable is quantified by their percentage increase in mean squared error (% IncMSE), thus quantifying the influence of a given variable on the measured NEE.

## 3 Results

Here we present carbon fluxes (NEE, GPP, and *R*_eco_) from the first year of *mxg* growth using a field at the SABR farm planted at a planting density (~80,000 rhizomes ha^−1^) commonly used in commercial *mxg* plantings in the US. We then compare these fluxes with those from the UIEF site which was planted with legacy technology and a three‐fold lower planting density. To interpret these carbon fluxes, we also present the influence of climatic variables and compare the carbon uptake strength of our “corn‐belt” sites to other *mxg* sites.

Carbon fluxes are reported from July 8 to December 31, 2008, for the UIEF site and from June 1 to December 31, 2019, for the SABR site, while climatological conditions are presented for the entire calendar year (2008 for UIEF and 2019 for SABR) in which the crop was established. The growing season was from June to September for both sites. We describe these weather conditions first to inform the reader of the environmental conditions during the critical crop emergence and establishment period.

### 3.1 SABR Weather Mirrored Long‐Term Trends, With Colder Winter *T*_air_

**FIGURE 1:**
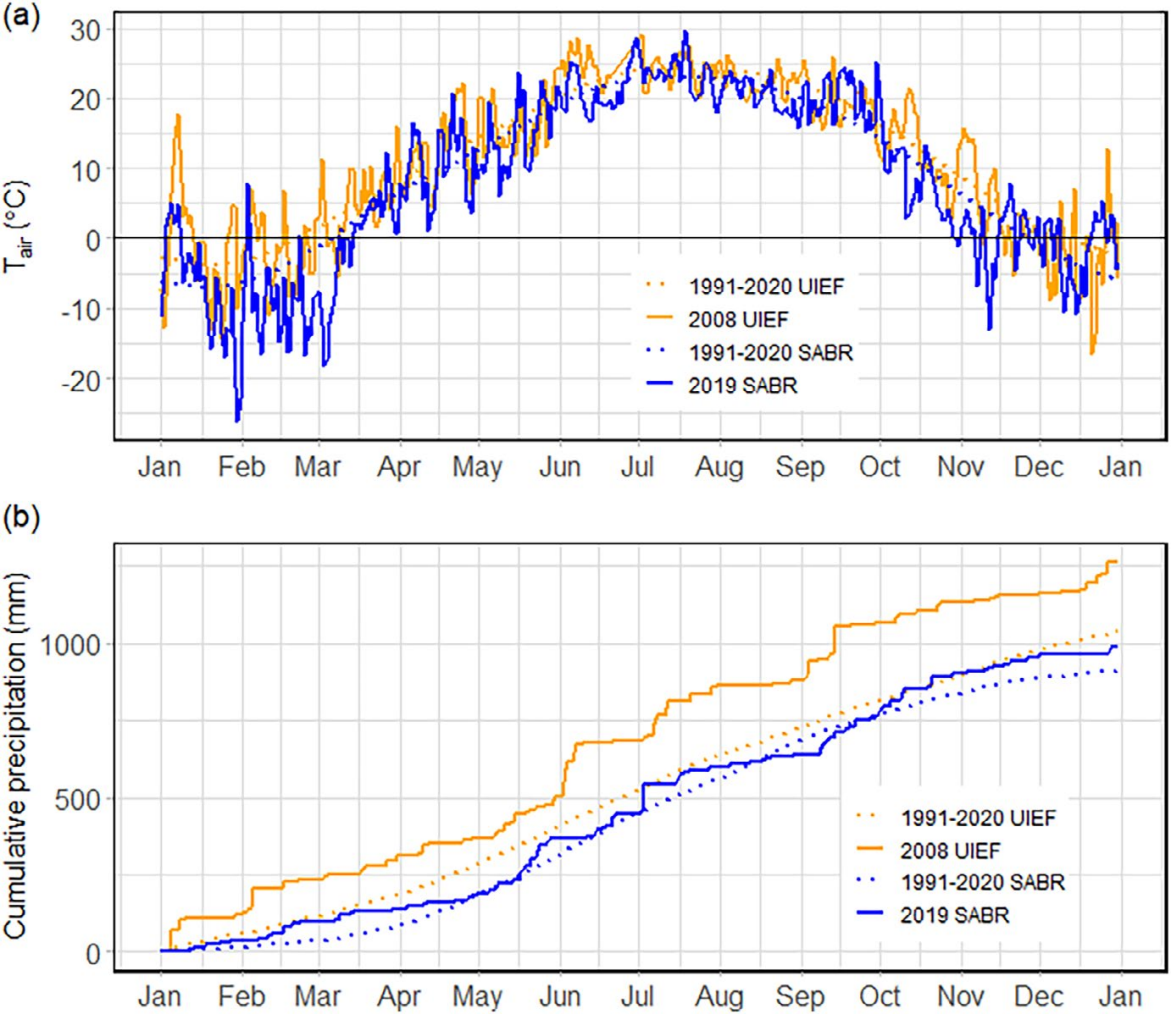
(a) Mean daily air temperature for Sustainable Advanced Bioeconomy Research Farm, Ames, IA, USA (SABR) in 2019 (solid blue line) and University of Illinois Energy Farm, Urbana, IL, USA (UIEF) in 2008 (solid orange line) against their respective climatological averages (1991–2020; dashed lines). (b) Cumulative annual precipitation for SABR (2019) and UIEF (2008) compared to the long‐term average (1991–2020). Solid lines represent measured daily mean climate data for SABR (blue, 2019) and UIEF (orange, 2008), while dotted lines indicate long‐term climate data (orange for UIEF, blue for SABR). Long‐term climate records sourced from NOAA (2023).

**FIGURE 2:**
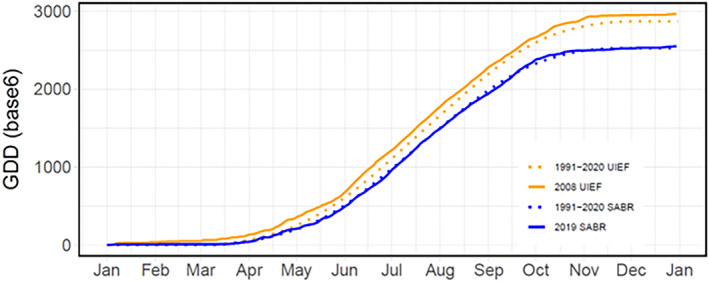
Total accumulated growing degree days (GDD, base 6) of *mxg* for SABR (2019, blue line) and UIEF (2008, orange line) sites. University of Illinois Energy Farm, Urbana, IL, USA (UIEF). Sustainable Advanced Bioeconomy Research farm, Ames, IA, USA (SABR).

In 2019, the SABR annual precipitation and daily mean T_air_ were consistent with the long‐term trend observed from 1991 to 2020 (Figure 1a,b). The average annual precipitation over the long term (1991–2020) is 912 ± 1.4 mm, with T_air_ ranging from a winter daily average low of −7.1°C to a summer high of 23.1°C (Figure 1a). While there were several days with extreme cold temperatures during the winter, these occurred prior to planting at SABR (Figure 1a). The last spring frost and first fall frost occurred within a week of their respective climatological averages. Overall, the 2019 growing season was slightly cooler than the long‐term (1991–2020) and the total growing season and annual precipitation were both slightly higher than average (Table 5). This increase in precipitation can be attributed in part to two significant precipitation events in late May and early July (Figures 1b and 2).

**TABLE 5:** Cumulative values for the growing season and the whole period. The data were available starting July 8 for UIEF, June 1 for SABR, so the growing season is between July 8 and September 30 for UIEF, June 1 and September 30 for SABR. The whole period was between July 8 and December 31 for UIEF, June 1 and December 31 for SABR. The percentage increase (%) shows how much higher one value is compared to another relative to the smaller value. University of Illinois Energy Farm, Urbana, IL, USA (UIEF). Sustainable Advanced Bioeconomy Research farm, Ames, IA, USA (SABR).

Variable	Growing season		Whole period		
	SABR	UIEF	SABR	UIEF	%
NEE (g C m^−2^)	−574	−204	−621	−198	214
GPP (g C m^−2^)	1295	650	1475	785	88
*R*_eco_ (g C m^−2^)	721	445	854	587	46
*R*_eco_/GPP	0.56	0.69	0.58	0.74	—
Precipitation (mm)	392	560	988	1262	28
GDD	1871	1304	2548	2964	16

### 3.2 SABR *Mxg* Showed Strong Carbon Sink

**FIGURE 3:**
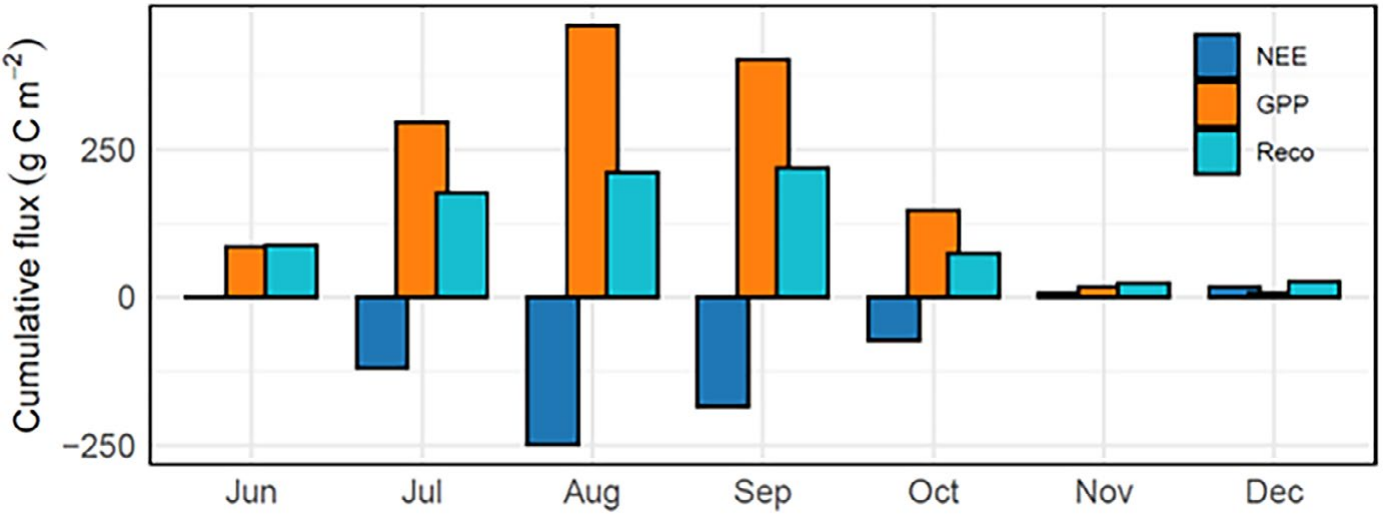
Monthly cumulative carbon fluxes, net ecosystem exchange (NEE), gross primary productivity (GPP) and ecosystem respiration (*R*_eco_) (g C m^−2^) at SABR in 2019. Sustainable Advanced Bioeconomy Research farm, Ames, IA, USA (SABR).

**FIGURE 4:**
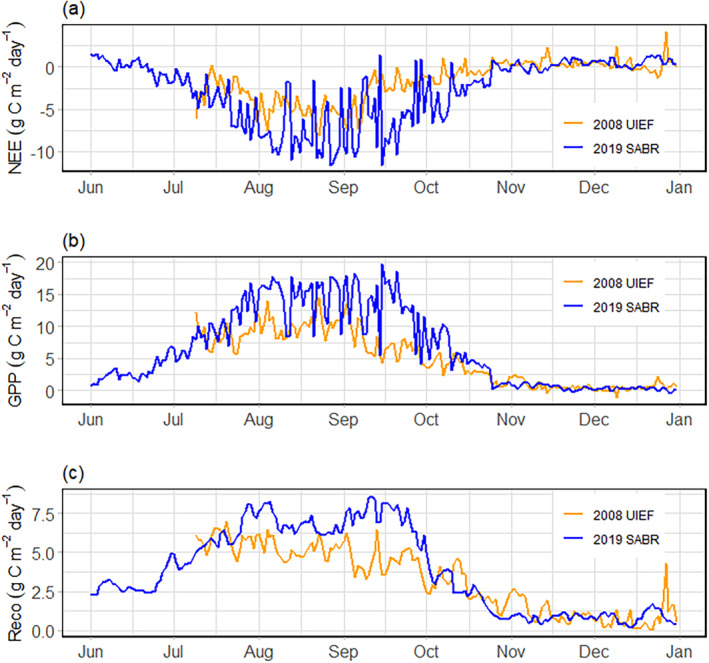
Daily mean (a) net ecosystem exchange (NEE), (b) gross primary productivity (GPP), and (c) ecosystem respiration (*R*_eco_), g C m^−2^ day^−1^, fluxes for UIEF (2008, orange line) and SABR (2019, blue line). University of Illinois Energy Farm, Urbana, IL, USA (UIEF). Sustainable Advanced Bioeconomy Research farm, Ames, IA, USA (SABR).

**FIGURE 5:**
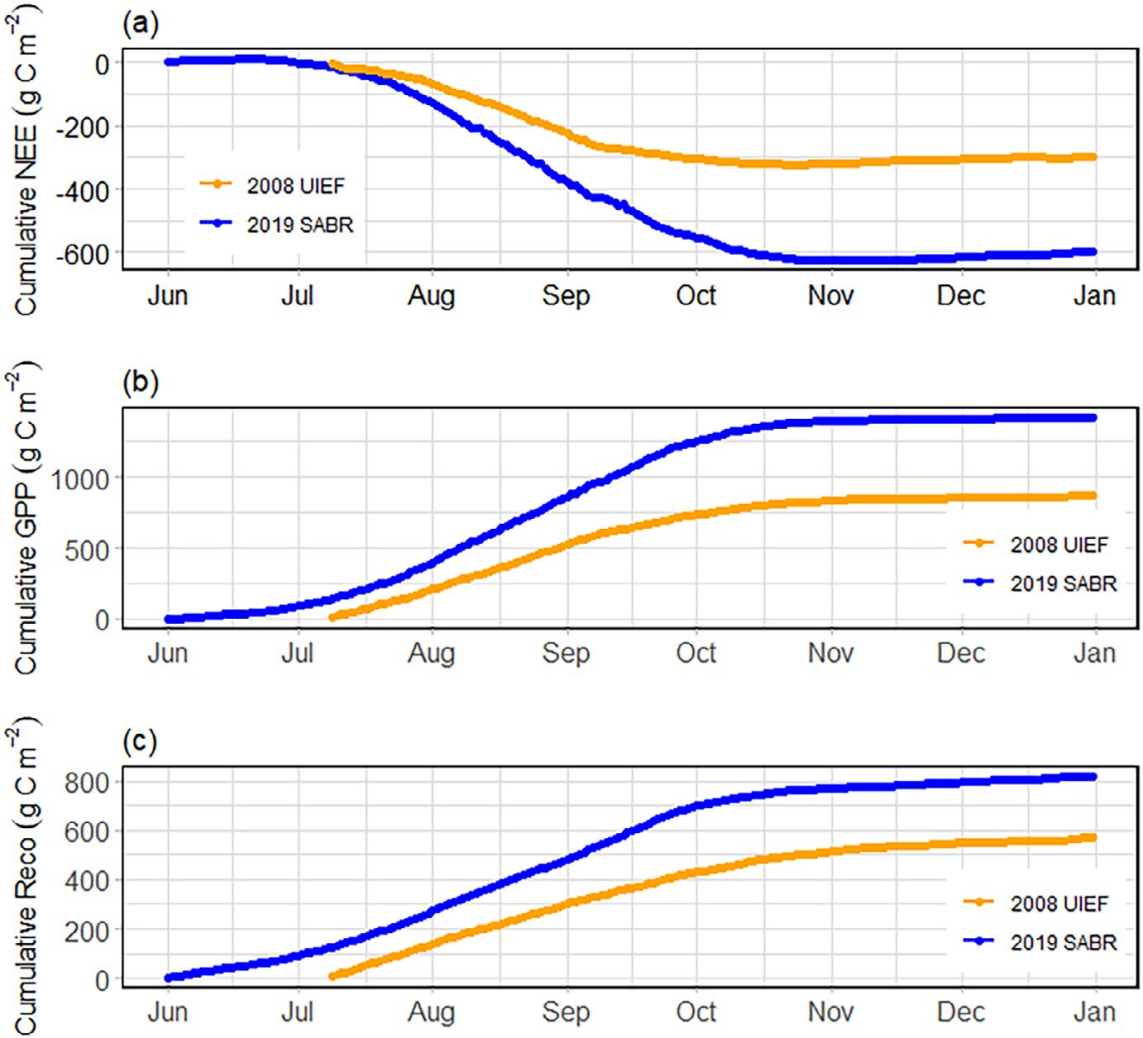
Cumulative (a) net ecosystem exchange (NEE), (b) gross primary productivity (GPP), and (c) ecosystem respiration (*R*_eco_), g C m^−2^, for UIEF (2008, orange line) and SABR (2019, blue line). University of Illinois Energy Farm, Urbana, IL, USA (UIEF). Sustainable Advanced Bioeconomy Research farm, Ames, IA, USA (SABR).

Overall, *mxg* at SABR showed a strong carbon uptake indicated by a prolonged period with large negative daily and monthly values resulting in a large negative cumulative NEE (Figures 3, 4, 5). Peak GPP values during the first growing season at SABR were 20 g C m^−2^ day^−1^, leading to a cumulative GPP of 1475 g C m^−2^ by the end of the establishment year. Cumulative *R*_eco_ during the first year was 854 g C m^−2^, leading to a cumulative NEE of −621 g C m^−2^ (Figure 5).

As the season progressed at SABR, GPP increased substantially, reaching its peak in August, while *R*_eco_ had a more moderate rise over the same time (Figure 3). This discrepancy led to the highest negative cumulative NEE in August (−248 g C m^−2^) and persisted into September (−183 g C m^−2^). The onset of senescence at the beginning of October corresponded with a swift decline in GPP, which led to diminished carbon uptake by the end of October. From November until the year's end, the ecosystem transitioned to a weak carbon source, with *R*_eco_ exceeding GPP (Figure 3).

For the growing season, the ratio of cumulative *R*_eco_ to cumulative GPP was approximately 0.56, indicating that photosynthesis was dominant over respiration and more carbon was assimilated than was released.

### 3.3 UIEF Had Significantly Higher Precipitation Than SABR, but Both Sites Experienced Similar, Long‐Term *T*_air_

The UIEF and SABR sites (Table 2) had comparable temperature conditions during the respective establishment seasons but had a notable difference in precipitation (Figure 1). The cumulative annual precipitation was 28% higher at UIEF (1262 ± 10.8 vs. 988 ± 6.8 mm at SABR; Figure 1b). This annual precipitation was considerably higher than UIEF's long‐term average (1991–2020, 1039 ± 0.7 mm), whereas SABR's annual precipitation was more consistent with its long‐term average (1991–2020, 912 ± 1.4 mm). Similarly, the growing season cumulative precipitation was 30% higher at UIEF (560 vs. 392 mm; Figure 1b, Table 5). Much of this rain fell in a significant precipitation event in early June at UIEF, leading to ponding which delayed and extended the planting period to June 2–16, 2008 (Figure 1b). By contrast, precipitation was 55% lower at SABR in June of 2019 compared to June of 2008 at UIEF; however, SABR precipitation was at or above the climatological average, a trend that persisted throughout the year (Figure 1b). The precipitation at SABR was highest in July and lowest in November, following a different pattern than observed during the UIEF establishment year (Figure 1b).

In the initial weeks after planting, both sites had similar *T*_air_ that were consistent with long‐term means (Figure 1a). The similarities in air and *T*_soil_ between the sites continued throughout the growing season. *T*_air_ and *T*_soil_ at SABR showed a similar trend with peaks in mid‐July (*T*_air_: 29.5°C, *T*_soil_: 32.1°C) and lows in December (*T*_air_: −13.1°C, *T*_soil_: −1.32°C; Figure 1a, Figure S1, Table 6).

**TABLE 6:** Daily mean with standard deviation (SD) values for the growing season. The data were available starting July 8 for UIEF, June 1 for SABR, so the growing season is between July 8 and September 30 for UIEF, June 1 and September 30 for SABR. University of Illinois Energy Farm, Urbana, IL, USA (UIEF). Sustainable Advanced Bioeconomy Research farm, Ames, IA, USA (SABR).

Variable	Growing season daily mean			
	SABR		UIEF	
	Mean ± SD	Max/Min	Mean ± SD	Max/Min
NEE (g C m^−2^ day^−1^)	−4.5 ± 3.8	−10.7/1.5	−2.3 ± 2	−7.1/1.4
GPP (g C m^−2^ day^−1^)	10.2 ± 5.5	18.5/0.8	7.5 ± 2.3	13.3/3.9
*R*_eco_ (g C m^−2^ day^−1^)	5.7 ± 1.9	8.4/2.3	5.1 ± 0.7	6.6/3.5
Air temperature (°C)	21.3 ± 3.1	27.5/15.5	21.8 ± 3.1	26.4/15.4
Soil temperature (°C)	24.3 ± 3.8	30.1/16.6	23.6 ± 3.2	29.7/18.5
Precipitation (mm)	3.7		5.1	
GDD	669.5		718.6	

### 3.4 SABR Carbon Uptake Was Three Times Higher Than UIEF Due to Greater GPP

Relative to *mxg* at UIEF, NEE and GPP at SABR were approximately three‐ and two‐fold higher during the establishment year, respectively (Figure 5, Tables 5 and 6). At SABR, peak and mean NEE and GPP values exceeded those observed at UIEF, despite similar R_eco_ rates between the sites. The differences in carbon fluxes between sites were a product of both greater average uptake each day, as well as greater peak amounts on days most suitable for growth (Table 5). Overall, the difference in cumulative NEE was largely because of the 88% higher cumulative GPP value at SABR given that the difference in cumulative R_eco_ only 46% greater (Table 5).

During the most active portions of the growing season (June–September), mean NEE and GPP values also differed between the two sites. During this period, the *mxg* at SABR had a larger mean NEE (−4.5 g C m^−2^ day^−1^) and mean GPP (10.2 g C m^−2^ day^−1^) compared to UIEF (−2.3 and 7.5 g C m^−2^ day^−1^, respectively) (Table 6). However, there was a similarity in mean R_eco_ values during the same period (5.7 and 5.1 g C m^−2^ day^−1^ for UIEF and SABR, respectively; Table 6).

In addition to the differences in carbon fluxes, yield data from both sites also indicate a substantial difference, with SABR showing significantly higher biomass yields than UIEF during the establishment year. The first‐season hand‐harvested yield was significantly higher at SABR, with 16.27 Mg ha^−1^ compared to just 0.9 Mg ha^−1^ at UIEF (Kantola et al. 2022).

**FIGURE 6:**
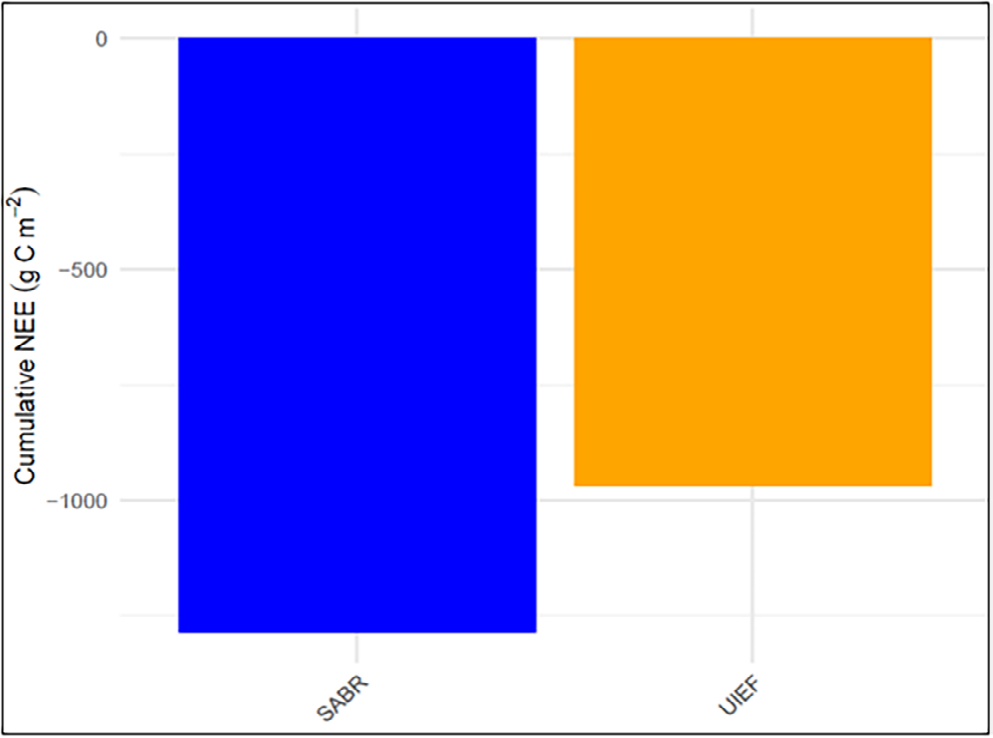
Cumulative net ecosystem exchange (NEE), g C m^−2^, for the establishment phase (3 years) at UIEF (July 2008–December 2011, orange bar) and SABR (June 2019—December 2021, blue bar). University of Illinois Energy Farm, Urbana, IL, USA (UIEF). Sustainable Advanced Bioeconomy Research farm, Ames, IA, USA (SABR).

We have also presented longer‐term cumulative NEE data for both UIEF and SABR to better understand carbon sink differences over time. Specifically, we compared the cumulative NEE after the first 3 years of establishment. The difference in the cumulative NEE observed between SABR and UIEF in the establishment phase, with SABR exhibiting a significantly higher carbon sink. SABR's cumulative NEE reached −1286 g C m^−2^, approximately 35% higher than UIEF's −970 g C m^−2^, indicating a sustained, but converging carbon uptake potential of the SABR site relative to UIEF after ~3 years of the establishment phase (Figure 6).

We additionally included the cumulative NEE for maize grown at both sites during the same years in Figure S4. This supplementary data is provided to illustrate how closely maize NEE values aligned between the two sites (cumulative NEE −518 and −550 g C m^−2^ for UIEF and SABR, respectively), offering a rough comparison to highlight the differences observed in *mxg* NEE between SABR and UIEF.

### 3.5 T_air_ and SWC Effects on NEE Slightly Higher in SABR Than UIEF

We found higher negative NEE (indicating a high carbon sink), higher GPP, and lower precipitation during the active growing season (June to September) in SABR than UIEF site‐years, while *R*_eco_, *T*_air_, *T*_soil_, and GDD showed similarity (Table 6).

**FIGURE 7:**
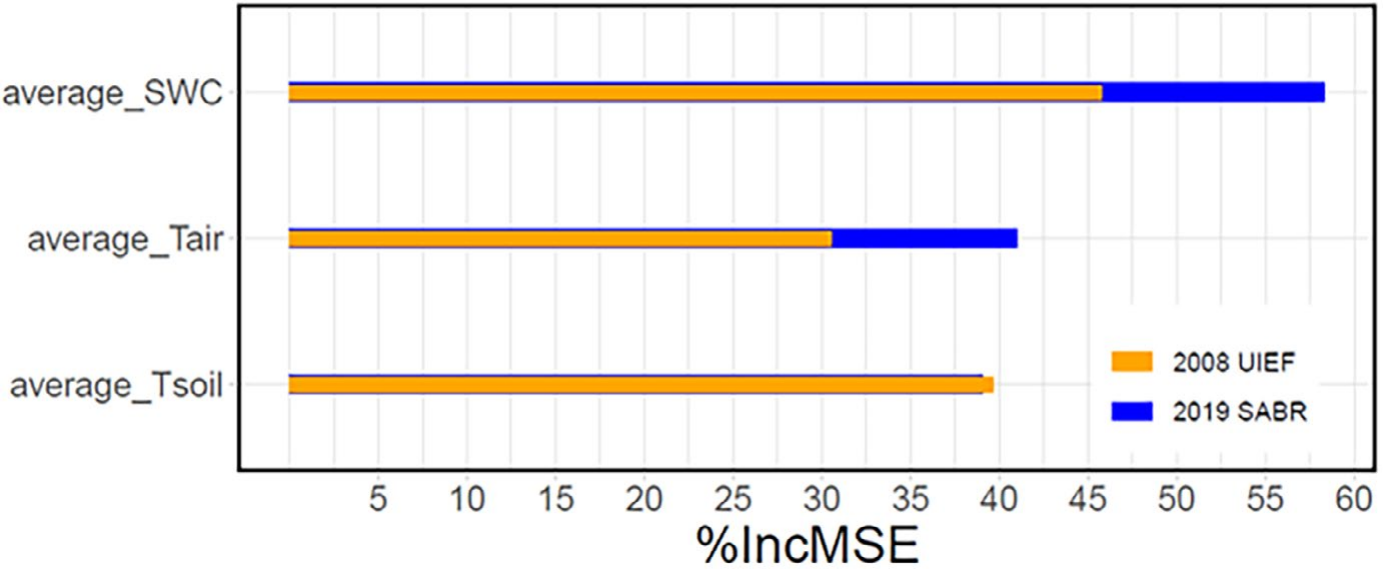
The influence of ecosystem variables: Average soil temperature (average_Tsoil), average air temperature (average_Tair), and average soil water content (average_SWC) on estimates of net ecosystem exchange (NEE) for the whole period was analyzed separately for UIEF and SABR using Random Forest analysis. The whole period was between June 1 and December 31 for SABR, July 8 and December 31 for UIEF. Percentage Increase in Mean Squared Error (%IncMSE) shows the relative importance of each variable. University of Illinois Energy Farm, Urbana, IL, USA (UIEF, orange line). Sustainable Advanced Bioeconomy Research farm, Ames, IA, USA (SABR, blue line).

The Random Forest analysis identified that SWC played a more significant role (by the highest percent increase in mean squared error; % IncMSE) in influencing NEE compared to *T*_air_ and *T*_soil_ at both sites, although the strength of these relationships varied (Figure 7). The *T*_air_ and SWC were slightly higher % IncMSE, at SABR relative to UIEF.

## 4 Discussion

Reducing uncertainty in estimates of the global carbon sequestration potential of biomass cropping systems requires observations under a range of agronomic and environmental conditions. In this study, we used the EC approach to quantify carbon fluxes of the perennial biomass crop, *mxg*, during the establishment year growing season that was recently planted in central Iowa (i.e., SABR), following commercial planting practices common in the US “corn‐belt.” We then compared our results to those of the most proximal (in both space and time) establishment of *mxg* that was also monitored using EC, namely UIEF in Central IL. Our key finding was that the cumulative annual NEE measured at the more recently established SABR site was nearly threefold greater than UIEF. Because carbon fluxes are driven by a complex combination of biophysical and management factors it is challenging to isolate the key difference between these sites. Here we attempt to provide insight on the factors that may lead to differing amounts of ecosystem carbon exchange by investigating our results and the conditions that may have led to them in the context of data available from other *mxg* sites monitored by EC around the globe.

### 4.1 Strong Carbon Uptake of *mxg* at SABR

**FIGURE 8:**
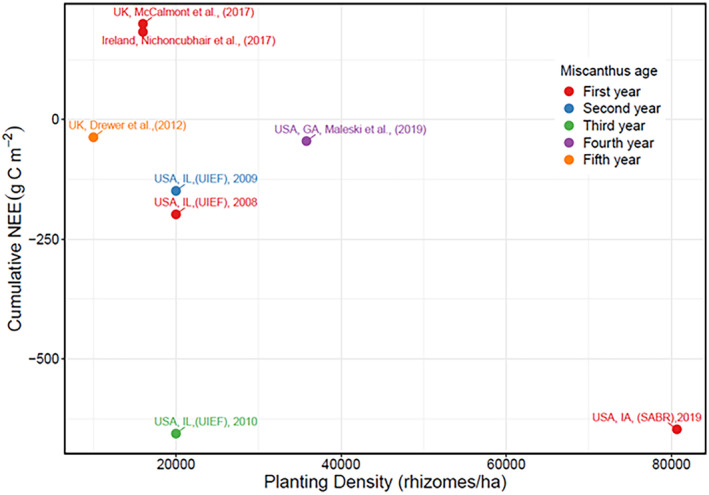
Cumulative net ecosystem exchange (NEE) values for *mxg* at different ages and planting densities as reported in various studies, including data from the first year of establishment in our study (SABR). University of Illinois Energy Farm, Urbana, IL, USA (UIEF). Sustainable Advanced Bioeconomy Research farm, Ames, IA, USA (SABR).

During the establishment phase, *mxg* at SABR displayed a large carbon uptake capacity of −621 g C m^−2^, which is several fold larger than previously reported first‐year values (Figure 4), and similar to an established *mxg* stand in its third growing season (Zeri et al. 2011) (Figure 8). This first‐season NEE for a more densely planted *mxg* field could drastically decrease the amount of time to offset the carbon losses associated with tillage and field preparations thus increasing the sustainability of *mxg* production. While it is likely that long‐term carbon uptake will plateau at a similar level of other established *mxg* (only so many plants can grow per unit area), the establishment season NEE has been shown to be quite variable, and management practices that drive NEE towards net carbon uptake could alleviate negative environmental impacts from land conversion.

In addition to the first‐season NEE benefits that were observed with the higher planting density at SABR, we also observed productivity increases as well. The first‐season hand‐harvested yield was found to be 16.27 Mg ha^−1^ at SABR, while hand harvested yields at UIEF were 0.9 Mg ha^−1^. Although the first‐year yields at SABR were less than the 27 Mg ha^−1^ observed at the UIEF in the third growing season, they were greater than the second year yield at UIEF which was 7.8 Mg ha^−1^ indicating that SABR *mxg* was nearly as productive as a third year stand of *mxg*. *Mxg* is rarely harvested in the establishment year due to low yields; however, the yields resulting from higher planting densities at SABR allowed for a first season harvest, which if repeatable, could have major impacts on the economics of *mxg* which should be further investigated.

Considering more than 1400 site years of EC data, Baldocchi (2014) reported a mean annual NEE of −156 ± 284 g C m^−2^ year^−1^ across a global range of crops and ecosystems. Positioning the −621 g C m^−2^ year^−1^ observed at SABR among the values reported from Baldocchi (2014) demonstrates the rarity of such a strong carbon sink. Furthermore, we were unable to find any published data from commercial crops which demonstrated a stronger carbon sink than the *mxg* at SABR.

The growing season conditions throughout the establishment phase were highly conducive to growth, and both temperatures and precipitation were near long‐term averages (Figure 1a,b). Precipitation and soil moisture during the growing season are crucial for *mxg* to reach its full biomass yield potential (Cosentino et al. 2007; Heaton et al. 2010; Richter et al. 2008). Similarly, Clifton‐Brown and Lewandowski (2002) posited that *mxg* exhibits diminished growth potential under conditions of low precipitation or extended drought periods. The Random Forest analysis conducted in this study further highlighted that SWC had the most significant impact on NEE at both sites, reinforcing the importance of adequate soil moisture for carbon uptake in *mxg* stands. We speculate the favorable growing conditions at SABR enhanced the growth potential of a higher density planting to amplify GPP, without substantially increasing *R*_eco_ thus leading to high carbon uptake within the first growing season. These results may not be typical, and further investigation is warranted to directly assess the effect of higher planting density over a broad range of climatic conditions.

### 4.2 Comparison of Carbon Uptake Between SABR and UIEF

The comparative analysis of the UIEF and SABR sites (Table 2) reveals a substantial difference in carbon uptake in the establishment year (Figures 4, 5, 6 and 8). The daily mean NEE for *mxg* at SABR was nearly double that of UIEF (Figure 4). This difference was driven primarily by the strongly negative NEE observed at SABR, while the NEE observed at UIEF was similar to other EC values reported for *mxg* with comparative planting densities (Table 1).

Although the observed annual NEE at UIEF was similar to other EC studies, environmental conditions very likely negatively impacted the carbon uptake potential. Comparing the establishment season to the long‐term average, there was significant precipitation at the UIEF during the planting window. These weather events delayed planting until June 2, 2008, and extended the planting window until June 16, 2008 (Zeri et al. 2011). Typically, *mxg* is planted in early May, thus the later planting shortened the growing season. Consequently, the UIEF *mxg* did not initiate carbon uptake until midsummer (Zeri et al. 2011). While NEE at the UIEF was similar to other studies, NEE at UIEF under more ideal growing conditions may have increased rates.

Despite NEE differences between SABR and UIEF, the trend in NEE was dominated by a higher difference in GPP at both sites (Tables 5 and 6). This finding is align with previous work that indicates a dominant role of GPP over R_eco_ in influencing NEE variations (Baldocchi 2014; Luyssaert et al. 2007; Marcolla, Rödenbeck, and Cescatti 2017; Moreaux et al. 2020; Wohlfahrt et al. 2008). However, it is important to note that this relationship may vary depending on site‐specific factors such as vegetation type, biomass residues, and soil carbon content. The observation that excess precipitation and delayed planting at UIEF led to reduced carbon uptake is consistent with prior research. Studies like those by Bao et al. (2022) and Shi et al. (2023) have demonstrated that variations in GPP can significantly impact an ecosystem's carbon balance. These variations may result from factors such as environmental conditions, management practices, or disturbances.

The comparison of SABR and UIEF highlights the effects of both management (planting density) and environmental conditions on *mxg* carbon uptake potential. SABR employed cutting‐edge planting technology that allowed for higher planting density than previously reported. In contrast, UIEF was established more than a decade ago using legacy planting techniques which were coupled with challenging planting conditions. The combination of increased planting density and more suitable planting conditions coincided with a threefold increase in carbon uptake relative to UIEF which had previously shown the most NEE during establishment year of *mxg* according to our literature review (Figure 8). The longer‐term data indicates that the SABR site had higher carbon uptake than the UIEF site by the end of the three‐year establishment phase; however, the rates of NEE appear to get more similar over time (Figure 6). While this supports our findings, which focused primarily on the establishment year where climatic and management factors were explained, extending the analysis to longer‐term data would provide a more comprehensive understanding. Future studies should consider interannual variations and other climatic influences over time to better capture the full carbon dynamics between the sites.

To further contextualize these findings, the cumulative NEE for maize grown at both sites during the same years showed similar values, indicating consistent carbon uptake under comparable management practices. This highlights that the substantial differences in carbon uptake between SABR and UIEF for *mxg* are likely due to differences in planting density and other management practices specific to the perennial crop, rather than underlying site differences alone.

### 4.3 Evaluating SABR's Carbon Uptake in the Context of Other Published Studies

To contextualize the markedly different values reported at UIEF and SABR, we compared the cumulative NEE of *mxg* with that of other EC studies in the literature that reported planting densities (Figure 8). Our search found five sites across five locations including UK, Ireland, and US regions and spanning a five‐year window. Initial year cumulative NEE ranged from −198 to 200 g C m^−2^ while reported planting densities ranged from 10,000 to 35,800 units (Table 1). Sites that were planted with similar densities (16,000 rhizomes/ha), one in Wales and the other in Ireland, at the first year of establishment showed similar cumulative NEE and were shown to be a carbon source as 200 and 183 g C m^−2^, respectively (Table 1). However, these stands showed substantial evidence of poor establishment within the first season, and also were grassland conversions. The conversion from perennially non‐tilled soil to another crop which requires an intermediary tillage operation will very likely result in rapid losses of soil C in the early phases of the stand leading to less negative NEE or positive NEE as demonstrated in McCalmont et al. (2017) and Ní Choncubhair et al. (2017). In the mature years of monitoring, specifically the fourth and fifth years, the studies showed nearly neutral NEE, with values of −45 and −37 g C m^−2^ reported by Maleski et al. (2019) and Drewer et al. (2011), respectively. In contrast, the SABR site demonstrated a strong carbon sink capacity, roughly ten times greater than these studies, with −621 g C m^−2^ in the first year. While growing conditions were favorable for a successful establishment at SABR, using commercial equipment and modern commercial best practices also contributed to a higher planting density, which among controllable variables is one of the most significant management differences between SABR and all other previously reported *mxg* EC studies.

The NEE values observed at SABR are consistent with research that has shown planting *mxg* at greater densities will lead to greater shoot density (Olave et al. 2017). Olave et al. (2017) found that increasing rhizome planting density from 180 to 1620 kg ha^−1^ led to a ~2.5‐fold increase in number of stems per area and seven times more biomass in the first growing season when compared to the low‐density plots. While Olave et al. (2017) used three different‐sized rhizomes ranging from 25 to 225 g, a rhizome suitable for planting weighs 28 g (Mike Fulgham, AGgrowTech LLC personal communication). Assuming a rhizome suitable for planting weighs approximately 28 g (Mike Fulgham, AGgrowTech LLC personal communication), the Olave et al. (2017) study ranged from 6428 to 57,857 rhizomes ha^−1^. Using the same conversion, the planting density at SABR was approximately 2240 kg ha^−1^ and UIEF was approximately 560 kg ha^−1^. Similarly, Danatalos, Archontoulis, and Mitsios (2007) showed that increasing planting density from 6700 to 10,000 rhizomes ha^−1^ significantly increased biomass in the 4th and 5th growing seasons.

Comparing the SABR results with previously published EC studies showed a strong correlation between planting density and carbon uptake strength (Figure S5). When comparing SABR with other EC studies with differing planting densities (Table 1), it was found that SABR exhibited greater carbon uptake. Additionally, the relationship between planting density and cumulative NEE appeared linear despite a large gap in intermediate densities (Figure 8). This bolsters the importance of planting density as an important management‐related variable controlling the carbon uptake potential of establishing *mxg*.

While we cannot unequivocally attribute the increased carbon uptake at SABR to planting density alone, the planting density used was more than double that of any other EC sites in the literature, and threefold that of the regionally comparative *mxg* at the UIEF, strongly suggesting that planting density is a management decision that may have strong impact on *mxg* ecosystem carbon sink capacity. However, it is also important to note that the planting time—being a month later in UIEF—could have played a contributing role in these observations. Additionally, the cumulative NEE observed during the establishment phase (Figure 6) supports this idea by showing that SABR still exhibited approximately 1.5 times higher carbon uptake than UIEF. However, the fact that this rate was lower than in the establishment year at SABR points to interannual variability in carbon uptake at the site. This highlights the need to consider environmental factors such as climatic conditions and soil processes when interpreting these changes (Moore et al. 2021). These factors, along with planting density, can significantly impact carbon sequestration, emphasizing the importance of multi‐year studies to fully understand ecosystem responses.

To address the knowledge gaps identified in understanding the relationship between planting density and both yield and NEE in *mxg* cropping systems, it is imperative to initiate comprehensive observational experiments and develop predictive models. These studies should aim to empirically quantify the impact of varied planting densities on carbon uptake and biomass yield under different climatic and soil conditions. For instance, multi‐location field trials could be established to measure carbon fluxes and biomass accumulation at various densities and across a range of environmental settings. Concurrently, modeling efforts should be enhanced to simulate the interactions between planting density, growth conditions, and carbon dynamics. This dual approach would provide a robust dataset for refining models that predict the potential of *mxg* to offset greenhouse gases globally.

Furthermore, the economic implications of varying planting densities must not be overlooked. Higher densities require more rhizomes, thereby increasing the initial investment. It is crucial to conduct cost–benefit analyses to evaluate whether the increased costs associated with higher planting densities are economically justified by the corresponding increases in yield and carbon uptake. Such analyses should consider not only the direct costs and benefits but also the potential for long‐term environmental and economic sustainability. Our work suggests that planting four times more rhizomes leads to an immediate threefold increase in carbon uptake during the first year of establishment. However, future research should explore whether this trend continues in subsequent years as the stand matures, and how well the stand maintains its carbon balance at higher density over time. Additionally, it is important to investigate potential trade‐offs in planting at lower density to achieve higher yields later, as observed at UIEF, versus planting at a higher density to maintain high yields over the long term. The relationship between high yield and high carbon stocks warrants further exploration, as well as determining if there is a limit to planting density versus carbon gain. Furthermore, it is essential to consider the role of phenotype, shaped by the interaction of genotype (G), environment (E), and management practices (M), because this interaction crucially determines how different planting densities will impact carbon balance and yield. Addressing these questions through comprehensive studies will provide valuable insights into optimizing planting density for maximizing both carbon uptake and economic returns in *mxg* systems. However, it is crucial to specifically acknowledge the current lack of information regarding *mxg* resilience in different climates and soil conditions, particularly at increased planting densities. For example, in lower‐grade soils, it remains uncertain whether *mxg* yields can be maintained at higher densities without additional inputs or depleting soil resources. Additionally, there is a potential risk of increased vulnerability to foliar or other disease issues under these conditions. Therefore, it is essential that multi‐location trials not only focus on first‐year NEE but also measure broader sustainability metrics. This holistic approach will enable stakeholders to make informed decisions that balance ecological benefits with economic viability, ultimately contributing to the broader goals of sustainable agriculture and climate change mitigation.

## Author Contributions

**Guler Aslan‐Sungur (Rojda):** conceptualization, data curation, formal analysis, visualization, writing – original draft, writing – review and editing. **Nic Boersma:** conceptualization, writing – original draft. **Caitlin E. Moore:** data curation, formal analysis, writing – review and editing. **Emily Heaton:** conceptualization, funding acquisition, writing – review and editing. **Carl J. Bernacchi:** funding acquisition, writing – review and editing. **Andy Vanloocke:** conceptualization, funding acquisition, writing – review and editing.

## Conflicts of Interest

The authors declare no conflicts of interest.

## Supporting information


Data S1.

## Data Availability

The data that support the findings of this study are openly available in DataShare: the Open Data Repository of Iowa State University at https://figshare.com/s/21fdfad84fb10349278b.
