# Supplementary material for: Advances in Miscanthus × Giganteus Planting Techniques May Increase Carbon Uptake in the Establishment Year
Source: Glob Change Biol Bioenergy. 2024 Nov 28;17(1):e70012. doi: 10.1111/gcbb.70012 (PMC11604094; doi:10.1111/gcbb.70012)
Supplement: Supplementary file 1 — Data S1. [file GCBB-17-e70012-s001.docx]

Supplementary Information

*
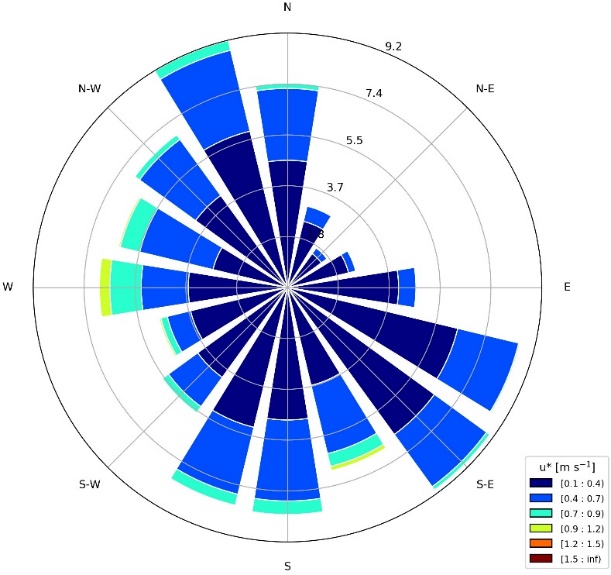
*
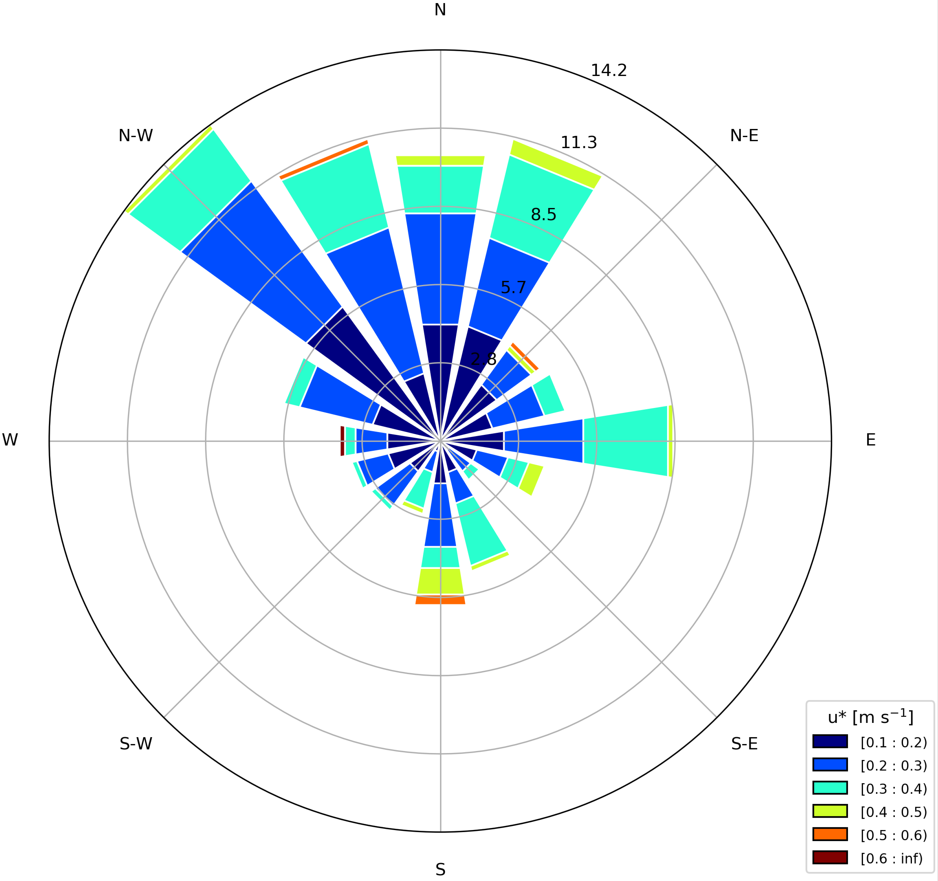

 (a) (b)

*Figure S1: Wind-rose map of the* Sustainable Advanced Bioeconomy Research farm, Ames, IA, USA (SABR) between June to September 2019 (a) and the University of Illinois Energy Farm, Urbana, IL, USA (UIEF) between July to September 2008 (b).


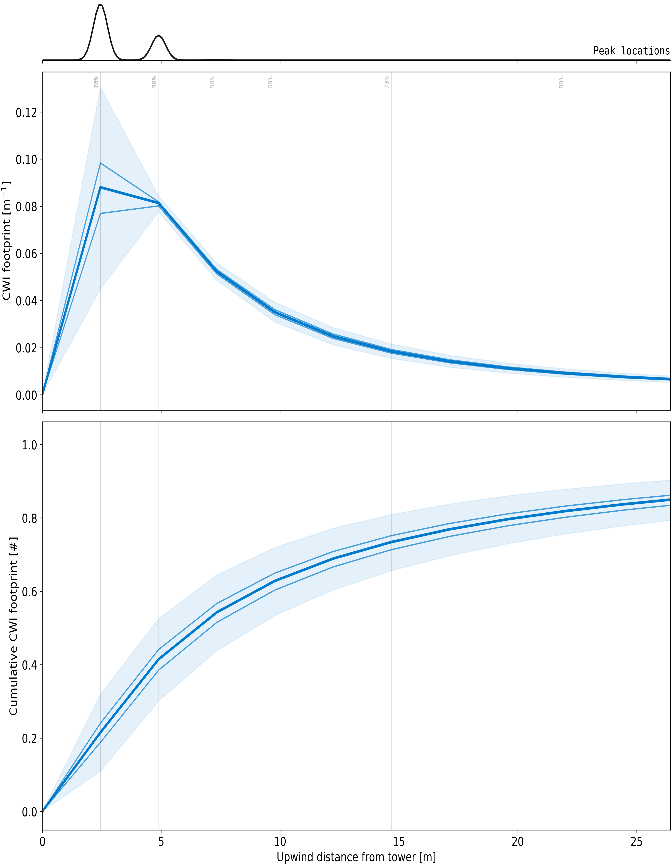

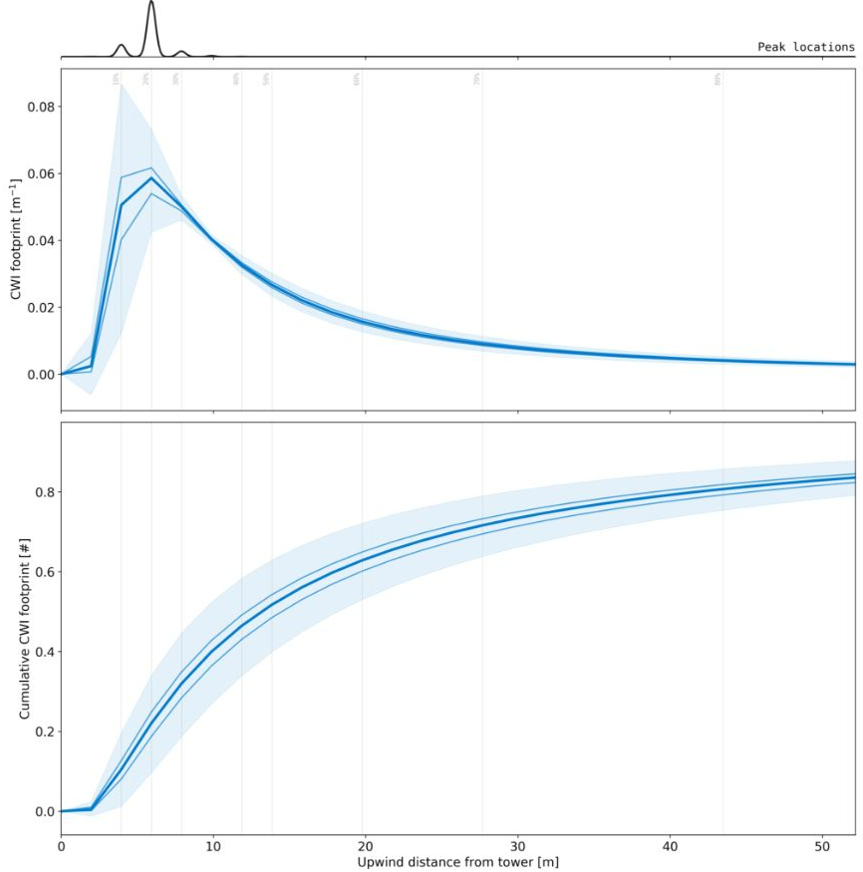


1. (b)

*Figure S2.* Footprint analysis for *mxg* at *the* Sustainable Advanced Bioeconomy Research farm, Ames, IA, USA (SABR) between June to September 2019 (a) and the University of Illinois Energy Farm, Urbana, IL, USA (UIEF) between July to September 2008 (b). Cross-wind integrated (CWI) footprint and Cumulative CWI (y-axis) versus Upwind distance from tower (m, x-axis) plots were exported using TOVI tool by Li-COR.


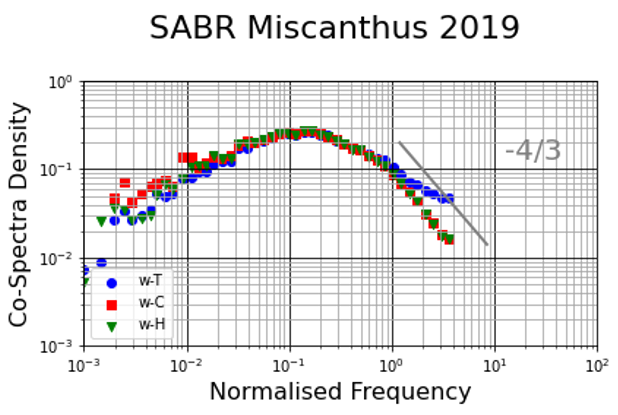


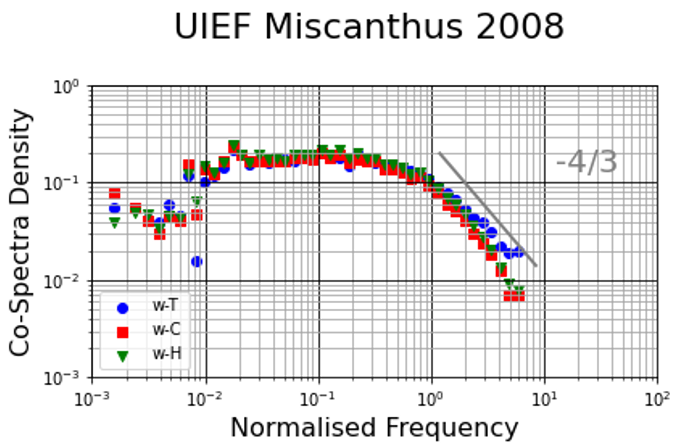

 (a) (b)

*Figure S3.* Cospectra plots for temperature (w-T), water (w-H) and carbon (w-C) against vertical wind (w) for an example 3 days period in the growing season (August 1^st^ to August 4^th^) without rainfall. The theoretical decay rate in the inertial subrange (-4/3) is indicated in grey as per Kaimal & Finnigan, (1994). Cospectra are binned into 50 exponentially spaced frequency groups and represent times of highest turbulent flux from 10:00 to 14:00 at time UTC-5:00. Sustainable Advanced Bioeconomy Research farm, Ames, IA, USA (SABR, a). University of Illinois Energy Farm, Urbana, IL, USA (UIEF, b).


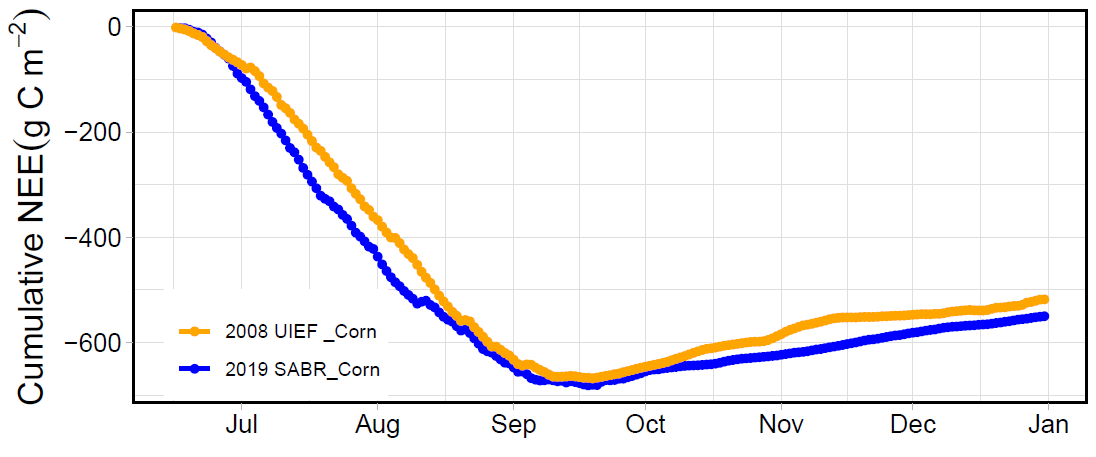


*Figure S4.* Cumulative net ecosystem exchange (NEE), g C m^-2^ of maize (corn) for UIEF (2008, orange line) and SABR (2019, blue line). University of Illinois Energy Farm, Urbana, IL, USA (UIEF). Sustainable Advanced Bioeconomy Research farm, Ames, IA, USA (SABR).


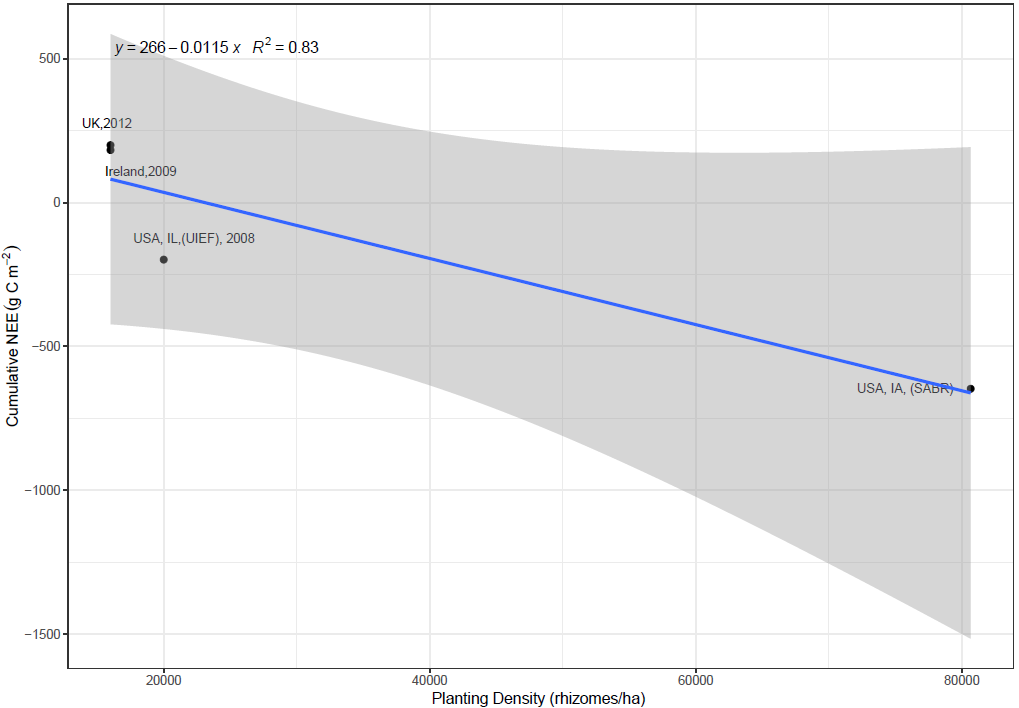


*Figure S5.* Cumulative Net Ecosystem Exchange (NEE) values for *mxg* at first year establishment and planting densities as reported in various studies, including data from the first year of establishment in our study (SABR). University of Illinois Energy Farm, Urbana, IL, USA (UIEF). Sustainable Advanced Bioeconomy Research farm, Ames, IA, USA (SABR). Blue line is the regression line with 95% confidence interval (gray shade). Regression coefficient (R^2^).

*Table S1.* Instrumentation installed on the flux towers at the Sustainable advanced bioeconomy farm, Ames, IA, USA (SABR) and University of Illinois Energy Farm, Urbana, IL, USA (UIEF).

| **Variable** | **Site** | **Instrument Model** | **Manufacturer** |
| --- | --- | --- | --- |
| CO_2_ & H_2_O | UIEF | Infrared gas analyzer, LI-7500/LI-7500A | LICOR Biosciences, Lincoln, NE, USA |
|  | SABR | Infrared gas analyzer, LI-7500DS |  |
| U,V,W, temperature | UIEF | 3-D sonic anemometer, 81000V/RE | R.M. Young, Traverse City, MI, USA |
|  | SABR | 3-D sonic anemometer, WindMaster | GILL, Hampshire, UK |
| Air Temperature,  Relative Humidity | UIEF | Aspirated probe, 43347-IX  HMP-45C | Campbell Scientific, Logan, UT, USA |
|  | SABR | HMP-155 |  |
| Wind Speed & Direction | UIEF | 2-D sonic anemometer, 85000V | R.M. Young, Traverse City, MI, USA |
|  | SABR | 3D sonic anemometer,  WindMaster | GILL, Hampshire, UK |
| Incoming and outgoing short and long wave radiation | UIEF | Four component radiometer, CNR1 | Kipp & Zonen, The Netherlands |
|  | SABR | Four component radiometer, CNR4 |  |
| Incoming and outgoing PAR | UIEF | Quantum sensor, LI-190 | LICOR Biosciences, Lincoln, NE, USA |
|  | SABR |  |  |
| Soil heat flux | UIEF | Heat flux plate, HFP01 | Hukseflux, The Netherlands |
|  | SABR | Self-Cal Heat Flux Plate, HFPO1SC |  |
| Soil moisture and temperature | UIEF | Hydra Probe II | Stevens Water Monitoring Systems, Inc., Portland, OR, USA |
|  | SABR |  |  |
| Data logging | UIEF | CR3000 | Campbell Scientific, Logan, UT, USA |
|  | SABR | SmartFlux 3 system | LICOR Biosciences, Lincoln, NE, USA |

Additional References

Kaimal, J. C., & Finnigan, J. J. (1994). *Atmospheric Boundary Layer Flows: their structure and measurement*. Oxford University Press.
